# Predictive Factors for *BRCA1* and *BRCA2* Genetic Testing in an Asian Clinic-Based Population

**DOI:** 10.1371/journal.pone.0134408

**Published:** 2015-07-29

**Authors:** Edward S. Y. Wong, Sandhya Shekar, Claire H. T. Chan, Lewis Z. Hong, Suk-Yean Poon, Toomas Silla, Clarabelle Lin, Vikrant Kumar, Sonia Davila, Mathijs Voorhoeve, Aye Aye Thike, Gay Hui Ho, Yoon Sim Yap, Puay Hoon Tan, Min-Han Tan, Peter Ang, Ann S. G. Lee

**Affiliations:** 1 Division of Medical Sciences, Humphrey Oei Institute of Cancer Research, National Cancer Centre, Singapore, Singapore; 2 Institute of Molecular and Cell Biology, Singapore, Singapore; 3 Cancer and Stem Cell Biology Program, Duke-NUS Graduate Medical School, Singapore, Singapore; 4 Human Genetics, Genome Institute of Singapore, Singapore, Singapore; 5 Department of Pathology, Singapore General Hospital, Singapore, Singapore; 6 Department of Surgical Oncology, National Cancer Centre, Singapore, Singapore; 7 Division of Medical Oncology, National Cancer Centre, Singapore, Singapore; 8 OncoCare Cancer Centre, Mount Elizabeth Novena Specialist Centre, Singapore, Singapore; 9 Department of Physiology, Yong Yoo Lin School of Medicine, National University of Singapore, Singapore, Singapore; 10 Office of Clinical and Academic Faculty Affairs, Duke-NUS Graduate Medical School, Singapore, Singapore; University of Hawaii Cancer Center, UNITED STATES

## Abstract

**Purpose:**

The National Comprehensive Cancer Network (NCCN) has proposed guidelines for the genetic testing of the *BRCA1* and *BRCA2* genes, based on studies in western populations. This current study assessed potential predictive factors for *BRCA* mutation probability, in an Asian population.

**Methods:**

A total of 359 breast cancer patients, who presented with either a family history (FH) of breast and/or ovarian cancer or early onset breast cancer, were accrued at the National Cancer Center Singapore (NCCS). The relationships between clinico-pathological features and mutational status were calculated using the Chi-squared test and binary logistic regression analysis.

**Results:**

Of 359 patients, 45 (12.5%) had deleterious or damaging missense mutations in *BRCA1* and/or *BRCA2*. *BRCA1* mutations were more likely to be found in ER-negative than ER-positive breast cancer patients (*P*=0.01). Moreover, ER-negative patients with *BRCA* mutations were diagnosed at an earlier age (40 vs. 48 years, *P*=0.008). Similarly, triple-negative breast cancer (TNBC) patients were more likely to have *BRCA1* mutations (*P*=0.001) and that these patients were diagnosed at a relatively younger age than non-TNBC patients (38 vs. 46 years, *P*=0.028). Our analysis has confirmed that ER-negative status, TNBC status and a FH of hereditary breast and ovarian cancer (HBOC) are strong factors predicting the likelihood of having *BRCA* mutations.

**Conclusions:**

Our study provides evidence that TNBC or ER-negative patients may benefit from *BRCA* genetic testing, particularly younger patients (<40 years) or those with a strong FH of HBOC, in Asian patients.

## Introduction

The National Comprehensive Cancer Network (NCCN) has recommended various guidelines for the genetic testing of *BRCA1* and *BRCA2*, which include specific criteria on the age at diagnosis of the patients and family members; the occurrence of breast, ovarian, pancreatic or prostate cancer in close relatives; and the diagnosis of triple-negative breast cancer (TNBC) [1]. Notably, TNBC patients have higher incidence rates of *BRCA1* and *BRCA2* mutations of up to 30% and 17% respectively [2–4], with younger TNBC patients (aged below 40 years) having an even higher incidence of 36% compared to those diagnosed below 50 years of 27% [5]. Most of these studies were based on Caucasian populations. It is unclear if these guidelines may also be adopted in Asian populations.

Next-generation sequencing (NGS) techniques enable the mutation screening of a larger set of samples in parallel, in a cost effective and accurate manner [6,7]. Recently, the emergence of NGS techniques has played an important role in the simultaneous screening of multiple cancer susceptibility genes including the *BRCA1* and *BRCA2* genes [8,9]. NGS technology has also been widely used in identifying novel genes with mutations related to HBOC [10,11].

Here, we studied 359 breast cancer patients to determine the prevalence of *BRCA* mutations in an Asian clinic-based population, using next-generation sequencing and Sanger sequencing. In addition, we evaluated the predictive value of ER-, PR- and HER2- receptor status, age at diagnosis, FH, and histological type for determining the likelihood of mutations in the *BRCA1* and *BRCA2* genes.

## Methods

### Patients

Peripheral blood samples were obtained from 359 breast cancer patients attending a risk assessment clinic at the National Cancer Centre Singapore (NCCS). Subjects were eligible if they had a FH of breast and/or ovarian cancer in first- and/or second-degree relatives (n = 176), or if they had early-onset breast cancer in the absence of FH (≤40 years of age) (n = 183). Patients were accrued from 2002 till 2013. Samples from two earlier studies (accrual from 1992 to 1996 and 2002 to 2006) were also included in this current study [12,13]. Of the 359 breast cancer patients, 321 (89.4%) were Chinese, 16 (4.5%) were Malays, 6 (1.7%) were Indians and 16 (4.5%) were of other Asian ethnicities. ER, PR and HER2 statuses were obtained from clinical databases, and were scored as positive or negative according to previously published criteria [14–16]; ER and PR were considered positive when nuclear staining was present in ≥1% of tumour cells. Her2 was considered as positive when >10% of tumour cells had strong (3+) cell membrane staining. The information for ER and TNBC status were available for 281 and 206 patients respectively. Written informed consent was obtained from all patients and the study was approved by the SingHealth Centralised Institutional Review Board.

### Mutational screening of *BRCA1* and *BRCA2*

S1 Fig shows a flow chart of the strategy used to detect mutations in the *BRCA1* and *BRCA2* genes, to predict damaging mutations and to identify driver/passenger mutations. Frameshift and nonsense mutations were considered to be deleterious.

Sanger sequencing of the *BRCA1* and *BRCA2* genes was performed as described previously [13], using the CEQ 8000 System (Beckman Coulter, Inc, CA, USA) or the ABI 3130 Genetic Analyzer (AB-Life Technologies; Thermo Fisher Scientific Corporation, MA, USA). The sequenced data were analyzed using the SeqMan Pro v.8.1.2 (Lasergene; DNASTAR, Madison, WI) software.

More recent DNA samples were sequenced by next-generation sequencing, either by SureSelect capture (Agilent Technologies Inc, CA, USA) followed by sequencing on the Illumina MiSeq platform, or SeqCap EZ capture (Roche Nimblegen, Basel, Switzerland) with sequencing on the Illumina HiSeq platform.

### Bioinformatic Analysis

For samples sequenced by NGS, reads were aligned to the UCSC human reference genome (hg 19) using the BWA aligner (version 0.5.6). Variant calling was done using the GATK Unified Genotyper [17], and CRISP pipelines [18] (for HiSeq).

All mutations identified from Sanger sequencing or NGS were annotated using the ANNOVAR tool, which provides tools such as SIFT, PolyPhen- II HDIV, PolyPhen—II HVAR, LRT and Mutation Taster to predict the effect of amino acid substitution for each missense mutation. Every missense mutation was scored as damaging or benign with each of the five prediction tools. If the missense mutation was scored as damaging by three or more of the prediction tools, the mutation was classified as a ‘Damaging’ mutation and if less than three, the mutation was classified as ‘Benign’. S1 Table shows the scores for the predictions from the various tools. All missense mutations were also checked against the BIC (http://research.nhgri.nih.gov/bic/), HGMD (http://www.hgmd.org/) and ClinVar (https://www.ncbi.nlm.nih.gov/clinvar/) databases, and were regarded as ‘pathogenic’ if classified as such in two or more databases. All deleterious or pathogenic mutations detected were confirmed by re-sequencing the samples by conventional Sanger sequencing, as described above.

### Multiplex Ligation-dependent Probe Amplification (MLPA)

All DNA samples were screened for large genomic rearrangements by MLPA using the SALSA MLPA P002-C2 *BRCA1* and SALSA MLPA P045-*BRCA2* test kits, and validated using the MLPA P087 and P077 confirmation kits (MRC-Holland, Amsterdam, Netherland), respectively. The MLPA analyses were done by DNA fragment analysis on the ABI 3130 Genetic Analyzer and comparative analysis of samples using the Coffalyser freeware v.131123.1303 (MRC-Holland, AM, Netherland).

### Statistical Analysis

Statistical analysis was done using SPSS version 18.0.2 (SPSS IBM, Armonk, NY). The non-parametric test, i.e., Mann-Whitney U-test was used to compare the median age of the carriers and non-carriers. The Fisher’s exact test was used to determine significant associations between clinico-pathologic features and the *BRCA* mutation status. Binary logistic regression analysis was used to estimate the predictive effects of the significantly associated factors for predicting the probability of *BRCA* mutations. *P*-values of <0.05 were considered statistically significant.

## Results

### Mutations in the *BRCA1* and *BRCA2* genes

Deleterious mutations detected in *BRCA1* and *BRCA2* are listed in S1 and S2 Tables. Frameshift and nonsense mutations, splice-site errors and large genomic rearrangements were classified as deleterious (n = 33). S3 Table shows the list of damaging missense mutations identified. Eleven of 68 missense mutations were predicted to be damaging.

Of 359 patients, 45 (12.5%) had deleterious or damaging missense mutations in the *BRCA1* and/or the *BRCA2* genes. One patient (case 79) had two deleterious mutations, a *BRCA2* nonsense (c.5645C>A; p.S1882X) and a *BRCA1* splice-site error (IVS7-15del10) (S1 and S2 Tables). Two patients had the same *BRCA1* deleterious mutation (c.67_68delinsAG; p.E23Rfs*18).

Three novel *BRCA1* mutations, including one frameshift, one nonsense and one large genomic rearrangement (S2 Fig) were detected as well as 11 *BRCA1* mutations that have been previously identified (S1 Table) [7,13,19–24]. Eight novel *BRCA2* frameshift mutations were identified, together with 10 mutations previously reported (S2 Table) [13,22,23].

### Clinico-pathological characteristics and mutational status

Table 1 shows the clinico-pathological features of cases with and without *BRCA1* and *BRCA2* mutations. The median age at diagnosis for *BRCA* mutation carriers was slightly higher than for non-carriers (41 *vs* 38) although not statistically significant.

**Table 1:** Characteristics of 359 breast cancer patients by mutational status.

	Total	With Mutation	Without Mutation
	*n* = 359	*n* = 33	*n* = 326
**Age at Diagnosis (Years)**			
**Median (range)**	38 (19–76)	41 (20–60)	38 (19–76)
≤ 40 years	239		
> 40 years	120		
**Family History**			
Breast and Ovarian Cancer (HBOC)	43 (12.0%)	13 (39.4%)	30 (9.2%)
Breast Cancer (BC)	132 (36.8%)	13 (39.4%)	119 (36.5%)
Ovarian Cancer (OC)	1 (0.3%)	0 (0.0%)	1 (0.3%)
Early Onset Breast Cancer	183 (50.9%)	7 (21.2%)	176 (54%)
**Histology**			
Infiltrating Ductal Carcinoma (IDC)	259 (72.2%)	19 (57.6%)	240 (73.6%)
Infiltrating Lobular (ILC)	12 (3.3%)	1 (3.0%)	11 (3.3%)
Medullary (IMC)	12 (3.3%)	0 (0.0%)	12 (3.7%)
Others	40 (11.1%)	5 (15.2%)	35 (10.7%)
Unspecified	36 (10.1%)	8 (24.2%)	28 (8.6%)
**ER Status**	***n* = 281**	***n* = 26**	***n* = 255**
Positive	202 (72.0%)	13 (50%)	189 (74.1%)
Negative	79 (28.0%)	13 (50%)	66 (25.9%)
**PR Status**	***n* = 279**	***n* = 25**	***n* = 254**
Positive	177 (63.4%)	13 (52%)	164 (64.6%)
Negative	102 (36.6%)	12 (48%)	90 (35.4%)
**HER2 Status**	***n* = 206**	***n* = 20**	***n* = 186**
Positive	49 (23.8%)	0 (0%)	49 (26.3%)
Negative	157 (76.2%)	20 (100%)	137 (73.7%)
**Patients with ER, PR & HER2 Status**	***n* = 206**	***n* = 20**	***n* = 186**
TNBC	28 (13.6%)	8 (40.0%)	20 (10.7%)
Non-TNBC	178 (86.4%)	12 (60.0%)	166 (89.2%)

Among 359 patients, 43 (12%) had a FH of HBOC, 132 (37%) had a FH of breast cancer, 1 (0.3%) had a FH of ovarian cancer and 183 (50.9%) were early-onset breast cancer patients without a FH (Table 1). *BRCA* mutation carriers were more likely to have a FH of HBOC than non-carriers (39.4% vs 9.2%). Conversely, *BRCA* carriers were less likely to have early-onset breast cancer in the absence of FH as compared to non-carriers (21.2% vs 54%).

The most common histological type of breast cancer in our study was infiltrating ductal carcinoma (IDC), at 72.2%, followed by infiltrating lobular carcinoma (ILC) (3.3%) and medullary cancer types (3.3%) (Table 1). Only 1 patients with ILC had *BRCA* mutations and none of the medullary cases had *BRCA* mutations. In patients with IDC, the percentage of *BRCA* mutation carriers was higher at 57.6%, as compared to other histological types of breast cancer.

The percentages of ER-positive and ER-negative patients were 72% (202/281) and 28% (79/281) respectively. *BRCA* mutation carriers, were likely to be ER-Negative than non-carriers (50% *vs* 25.9%). All *BRCA* mutation carriers with known Her2 status had HER2 negative tumors. Of 206 patients with known ER, PR and HER2 status, 13.6% were TNBC patients. Among our 28 TNBC patients, eight (40%) were *BRCA* mutation carriers.

### Associations between *BRCA1* and *BRCA2* mutation status with ER or TNBC status

There was a significant association of ER-negativity with *BRCA1* mutation carriers (61.5% vs 26.5%, *P* = 0.01, (Table 2); however, no difference was observed in *BRCA2* mutation carriers compared to the non-carriers. Furthermore, ER-negative patients (8/79) were more likely to have *BRCA1* mutations than ER-positive patients (5/202) (10% *vs* 2.5%, *P* = 0.01).

**Table 2:** Association between ER status, TNBC status, with *BRCA* mutation status.

	*BRCA1*					*BRCA2*				
	Carriers		Non-*BRCA1* carriers		*P*-value*	Carriers		Non- *BRCA2* Carriers		*P*-value*
	N = 13	%	N = 268	%		N = 14	%	N = 267	%	
**ER-positive (n = 202)**	5	38.5	197	73.5		9	64.3	193	72.3	
**ER-negative (n = 79)**	8	61.5	71	26.5	**0.01**	5	35.7	74	27.7	0.546
	N = 11	%	N = 195	%		N = 9	%	N = 197	%	
**Non-TNBC (n = 178)**	5	45.5	173	88.7		7	77.2	171	86.8	
**TNBC (n = 28)**	6	54.5	22	11.3	**0.001**	2	22.8	26	13.2	0.352

*P-values that were statistically significant are indicated in **bold**.

Similarly there was a strong association between *BRCA1* carriers and TNBC status (54.5% vs 11.3%, *P* = 0.001, (Table 2). TNBC patients were more likely to have *BRCA1* mutations (6/28) than non-TNBC patients (5/178) (21.4% *vs* 2.8%, *P* = 0.001).

### Associations between clinical characteristics with ER or TNBC status

The median age at diagnosis for ER-positive and ER-negative patients was 40 years and 39 years respectively (Table 3). In addition, the age at diagnosis for ER-negative patients with *BRCA* mutations was significantly younger than for ER-positive patients (40 *vs* 48, *P* = 0.008). When stratified by *BRCA1* and *BRCA2* mutational status independently, age at diagnosis for ER-negative patients with *BRCA1* and *BRCA2* mutations was significantly younger than for ER-positive patients (39.5 *vs* 50, *P* = 0.053 and 40 *vs* 48, *P* = 0.031, respectively) (Table 3).

**Table 3:** Association between clinical characteristics of breast cancer patients with ER or TNBC status.

	ER Status				TNBC Status			
	Total *n* = 281	Positive *n* = 202 (72%)	Negative *n* = 79 (28%)	*P*-value*	Total *n* = 206	TNBC *n* = 28(13.6%)	Non-TNBC n = 178(86.4%)	*P*-value*
**Age at Diagnosis (Years)**		**Median(range)**	**Median(range)**			**Median(range)**	**Median(range)**	
		40 (22–76)	39 (19–65)	0.284		38 (22–65)	40 (19–74)	0.236
***BRCA***								
Non-carriers		38 (22–76)	38 (19–65)	0.480		37.5 (24–65)	39 (19–74)	0.481
Carriers		48 (29–60)	40 (22–52)	**0.008**		38 (22–52)	47 (29–60)	**0.03**
**Among carriers**								
*BRCA 1*		50 (35–57)	39.5 (22–52)	0.053		38 (22–52)	46 (43–57)	**0.028**
*BRCA 2*		48 (29–60)	40 (35–40)	**0.031**		38.5 (37–40)	48 (29–60)	0.359
**Family History**								
Breast and Ovarian Cancer (HBOC)	32 (11.4%)	21 (10.4%)	11 (13.9%)	0.408	29 (14.1%)	8 (28.6%)	21 (11.8%)	**0.035**
Breast Cancer (BC)	115 (40.9%)	87 (43.1%)	28 (35.4%)	0.281	96 (46.6%)	12 (42.9%)	84 (47.2%)	0.690
Ovarian Cancer (OC)	0 (0.0%)	0 (0.0%)	0 (0.0%)		0 (0.0%)	0 (0.0%)	0 (0.0%)	
Early Onset Breast Cancer	134 (47.7%)	94 (46.5%)	40 (50.6%)	0.596	81 (39.3%)	8 (28.6%)	73 (41.0%)	0.298
**Histology**								
Infiltrating Ductal Carcinoma (IDC)	224 (79.7%)	156 (77.2%)	68 (86.1%)	0.102	159 (77.2%)	22 (78.6%)	137 (77.0%)	1
Infiltrating Lobular (ILC)	10 (3.6%)	8 (4.0%)	2 (2.5%)	0.731	8 (3.9%)	1 (3.6%)	7 (3.9%)	1
Medullary (IMC)	12 (4.3%)	10 (5.0%)	2 (2.5%)	0.519	11 (5.3%)	0 (0.0%)	11 (6.2%)	0.367
Others	29 (10.3%)	23 (11.4%)	6 (8.0%)	0.393	24 (11.7%)	4 (14.3%)	20 (11.2%)	0.750
Unspecified	6 (2.1%)	5 (2.5%)	1 (1.3%)	1	4 (1.9%)	1 (3.6%)	3 (1.7%)	0.445

*P-values that were statistically significant are indicated in **bold**.

The median age at diagnosis for TNBC patients was younger than for non-TNBC patients although not statistically significant (38 *vs* 40) (Table 3). The median age at diagnosis for TNBC patients with *BRCA* mutations was significantly younger than for non-TNBC patients with *BRCA* mutations (38 *vs* 47, *P* = 0.03). When stratified by *BRCA1* or *BRCA2* mutational status independently, age at diagnosis for TNBC patients with *BRCA1* and *BRCA2* mutations was significantly younger than for non-TNBC patients (38 *vs* 46 and 38.5 *vs* 48, respectively, *P* = 0.028).

Furthermore, the percentage of TNBC patients with a FH of HBOC was higher than for non-TNBC (28.6% *vs* 11.8%, *P* = 0.035). However, there was no statistical difference between the TNBC and non-TNBC patients for patients with other FH.

### Predictive factors for *BRCA1* and *BRCA2* mutations in ER and TNBC patients

Table 4 shows the potential predictive factors for *BRCA1* and *BRCA2* mutation carriers determined by binary logistic regression analysis. The analyses showed that of all the clinico-pathological characteristics (ER status, age at diagnosis, pedigree diagnosis and histological data), ER-negative status and a FH of HBOC were the strongest predictors for *BRCA1* or *BRCA2* mutations. The likelihood of patients with HBOC having *BRCA1/2* mutations was higher than for other patients (Odds ratio [OR] 3.898; 95% confidence interval [CI] 1.518–10.011; *P* = 0.005). The odds of having a *BRCA1* or *BRCA2* mutation in ER-positive patients was 0.390 times (95% CI 0.172–0.887; *P* = 0.025) less than ER-negative patients (OR 2.562; 95% CI 1.127–5.826; *P* = 0.025). Neither of the beta coefficients of both factors exceeded the absolute constant value (1.890), indicating that a single factor was insufficient to predict the mutation status. Both ER negative status and a FH of HBOC are required to predict the likelihood of having a *BRCA1/2* mutation.

**Table 4:** Potential predictive factors for *BRCA1* and *BRCA2* mutations in patients stratified by ER status and TNBC status.

Factor	Beta	Standard Error	Odds ratio	95% C.I. for Odds ratio		*P–*value*
				Lower	Upper	
**ER status (n = 281)**						
Estrogen Receptor Status (Positive)	-0.941	0.419	0.39	0.172	0.887	**0.025**
Hereditary Breast and Ovarian Cancer (HBOC)	1.36	0.481	3.898	1.518	10.011	**0.005**
Constant	-1.89	0.334	0.151			**0.001**
**TNBC status (n = 206)**						
Triple Negative Breast Cancer (TNBC)	1.537	0.531	4.651	1.643	13.163	**0.004**
Hereditary Breast and Ovarian Cancer (HBOC)	1.152	0.548	3.164	1.08	9.268	**0.036**
Constant	-2.835	0.332	0.059			**<0.001**

*P-values that were statistically significant are indicated in **bold**.

Similar analyses were performed to investigate the potential contribution of PR status, as a predictive factor, to predict the likelihood of having *BRCA1/2* mutations. However, no statistical significance was found (data not shown).

A similar analysis was done to evaluate the potential predictive factors for *BRCA1* and *BRCA2* mutations carriers in TNBC patients (Table 4). The analyses showed that of all the clinico-pathological characteristics (including age of diagnosis, family history and histology), TNBC status and a FH of HBOC were the strongest predictors for *BRCA1* or *BRCA2* mutations. The likelihood of patients with HBOC being diagnosed with *BRCA1/2* mutations was 3.164 times higher than for other patients (OR 3.164; 95% CI 1.080–9.268; *P* = 0.036). The odds of TNBC patients being diagnosed with *BRCA1/2* mutations was 4.651 higher compared to non-TNBC patients (OR 4.651; 95% CI 1.643–13.163; *P* = 0.004). Similarly, neither of the beta coefficients of both factors exceeded the absolute constant value (2.835), indicating that a single factor was insufficient to predict the mutation status. Thus, both TNBC status and a FH of HBOC are required to predict the probability of having *BRCA1/2* mutations.

### Predictive factors for *BRCA1* and *BRCA2* mutations, with the inclusion of damaging missense mutations

Binary logistic regression analysis was performed as before but with deleterious *BRCA* mutations as well as damaging missense mutations (S4–S7 Tables). However, unlike the previous analyses, the median ages did not show any significant difference. Moreover, the analysis showed that only the FH of HBOC is necessary in patients with known ER status, to predict the likelihood of *BRCA* mutations (S7 Table). For patients with known TNBC status, having a TNBC status and a FH of HBOC are required as predictive factors for *BRCA* mutation testing (S7 Table).

## Discussion

There are few Asian studies that have evaluated the association of *BRCA* mutation status and clinical characteristics. This current Singapore study, based on 359 Asian breast cancer patients prospectively accrued from a risk-assessment clinic, has identified ER-negativity, TNBC status and a FH of HBOC as predictive factors to increase the likelihood of detecting *BRCA1* and *BRCA2* mutations.

Approximately 70–80% of *BRCA1*-associated breast cancer cases are ER-negative [25–29]. We found that *BRCA1* carriers are more likely to be ER-negative as has been reported previously in western populations [30]. ER-negative status has been suggested to be intrinsic to *BRCA1*-related cancer as it has been found that the proportion of ER-negative patients with *BRCA1* mutations was significantly higher than for ER-positive patients [31].

Patients with *BRCA* mutations were diagnosed at an earlier age in this study. *BRCA1*-associated breast cancers have been shown to be more likely ER-negative for each age group (<45, 45–54, and 55–64 years), with an increase in ER-positive breast cancers with increasing age [31]. Our data concurs with these findings. Furthermore, we provide evidence that ER-negative patients with either *BRCA1* or *BRCA2* mutations were significantly younger than ER-positive patients.

In our cohort, 14% (28/206) of our patients were TNBC, of which approximately 21.4% (6/28) and 7.1% (2/28) had *BRCA1* and *BRCA2* mutations, respectively. A higher frequency of *BRCA1* mutations (20.9%) as compared to *BRCA2* mutations (3.6%) was also observed in another study on TNBC patients from Malaysia [32]. A literature review by Pershkin *et al* has reported that among TNBC patients, the proportion of *BRCA1* and *BRCA2* carriers ranged from 9 to 100% and 2 to 12%, respectively [33].

Among our *BRCA1* mutation carriers, 37.5% (6/16) were TNBC patients; whilst among our *BRCA2* carriers, 10.5% (2/19) were TNBC patients. This frequency of *BRCA* mutations in our TNBC patients is slightly lower than that reported by Peshkin *et al* (2010) of between 42% to 100% and 14% to 35%, for *BRCA1* and *BRCA2* mutations respectively [33].

Our logistic regression analyses indicated that the odds ratio of TNBC patients with HBOC having *BRCA1/2* mutations was 3.164 (95% CI 1.080–9.268; *P* = 0.036), highlighting the importance of FH when estimating *BRCA* mutations prevalence. This is consistent with another study from the US that reported that TNBC patients with a FH of breast cancer or ovarian cancer had a higher probability of having *BRCA* mutations as compared to those without any FH of breast cancer or ovarian cancer (57% *vs* 29%; *p*<0.001 and 77% *vs* 41%; *p*<0.001, respectively) [34].

The NCCN guidelines have proposed the inclusion of TNBC patients aged 60 years or younger for *BRCA* mutation testing. Recently, a Korean study demonstrated that TNBC patients are more likely to be diagnosed at a younger age than non-TNBC patients in the cohort (42 *vs* 44.1) although the association was not statistically significant [35]. Nevertheless, in the mutation carriers, the mean age at diagnosis of TNBC patients was older than for the non-TNBC patients (39.2 *vs* 34.6 for *BRCA1* and 51.5 *vs* 44.0 for *BRCA2*). Our data, however, are in contrast to these findings. We showed that the median age at diagnosis for TNBC patients with either *BRCA1* or *BRCA2* is younger than for non-TNBC patients (38 *vs* 46 for *BRCA1* and 38.5 *vs* 48 for *BRCA2*), suggesting that *BRCA1-* and *BRCA2*-associated breast cancer is most likely early-onset. A study from Malaysia showed that TNBC patients aged below 35 years had a higher prevalence of *BRCA1* and *BRCA2* mutations compared to non-TNBC patients (28% *vs* 9.9%) [32]. However, additional studies in larger populations from Asia are warranted to verify these findings from Malaysia and Singapore.

Collectively, and confirming previous findings in western populations, our results showed that the likelihood of TNBC patients being diagnosed with *BRCA* mutations was higher compared to non-TNBC patients, and the inclusion of additional criteria like a FH of HBOC may increase the probability of identifying *BRCA1/*2 mutations. A study from Malaysia showed an improvement in the sensitivity and specificity of the Manchester scoring system with the combination of negative ER status, FH and TNBC status [32].

In conclusion, our data showed that almost half of our *BRCA* mutation carriers in our cohort, are ER-negative. We also found that 29% (8/28) of TNBC patients are *BRCA* mutation carriers, with the majority being *BRCA1* mutation carriers. In addition, we have shown that our TNBC patients with either *BRCA1* or *BRCA2* mutations were diagnosed at an earlier age. The discovery of the predictive factors, ER-negative status, TNBC status and HBOC, in our study, warrants confirmation in additional Asian populations.

## Supporting Information

S1 Fig Flow chart of the strategy used for the detection and analysis of mutations in the *BRCA1* and *BRCA2* genes. * Computational algorithms used were SIFT, Polyphen-II HDIV, Polyphen-II HVAR, LRT and Mutational Taster; ^#^ Filtration criteria is explained in the methods section.
(TIF)

S2 Fig Deletion of Exons 16 to 19 in *BRCA1*. A) Gel photo of PCR products obtained from the amplification of a 563bp-target region from the sample FH42 and control cDNA template; and a sequencing chromatogram of the 272bp-band observed from FH42. (B and C) Changes in mRNA sequence brought about by the deletion of 291bp in FH42 and its corresponding amino acid sequence.
(TIF)

S1 Table Deleterious mutations in *BRCA1*. C, Chinese; I, Indian; B, Burmese; M, Malay; BC, Breast Cancer; OC, Ovarian Cancer; PC, Pancreatic Cancer; IDC, Invasive Ductal Carcinoma; D&L, Mixed Ductal and lobular; ILC, Invasive Lobular Carcinoma; HR, Hormone Receptor; ER, Estrogen Receptor; PR, Progesterone Receptor; TNBC, Triple Negative Breast Cancer; Unk, Unknown; Fs, Frameshift; Del, Deletion of exon; Dup, Duplication of exon; N, Nonsense; SE, Splice-site Error; Ref, References.
(XLSX)

S2 Table Deleterious mutations in *BRCA2*. C, Chinese; I, Indian; B, Burmese; M, Malay; BC, Breast Cancer; OC, Ovarian Cancer; PC, Pancreatic Cancer; IDC, Invasive Ductal Carcinoma; D&L, Mixed Ductal and lobular; ILC, Invasive Lobular Carcinoma; HR, Hormone Receptor; ER, Estrogen Receptor; PR, Progesterone Receptor; TNBC, Triple Negative Breast Cancer; Unk, Unknown; Fs, Frameshift; Del, Deletion of exon; Dup, Duplication of exon; N, Nonsense; SE, Splice-site Error; Ref, References.
(XLSX)

S3 Table Damaging missense mutations in the *BRCA1* and *BRCA2* genes. Ca, Cancer; C, Chinese; M, Malay; BC, Breast Cancer; OC, Ovarian Cancer; IDC, Invasive Ductal Carcinoma; ILC, Invasive Lobular Carcinoma; DCIS, Ductal Carcinoma in situ; HR, Hormone Receptor; ER, Estrogen Receptor; PR, Progesterone Receptor; TNBC, Triple Negative Breast Cancer; Unk, Unknown; Ref, References; OS, Overall Scores for the prediction of damaging mutations; D, Damaging; P, Potential damaging; N, Neutral.
(XLSX)

S4 Table Characteristics of 359 breast cancer patients by mutational status. * P-values that were statistically significant are indicated in bold; NS: Not Significant.
(XLSX)

S5 Table Association between ER status, TNBC status, with *BRCA* mutation status. * P-values that were statistically significant are indicated in bold; NS: Not Significant.
(XLSX)

S6 Table Association between clinical characteristics of breast cancer patients with ER or TNBC status. * P-values that were statistically significant are indicated in bold; NS: Not Significant.
(XLSX)

S7 Table Potential predictive factors for *BRCA1* and *BRCA2* mutations in patients stratified by ER status and TNBC status. (XLSX)
