# Supplementary figures and images for: Predictive Factors for BRCA1 and BRCA2 Genetic Testing in an Asian Clinic-Based Population
Source: PLoS One. 2015 Jul 29;10(7):e0134408. doi: 10.1371/journal.pone.0134408 (PMC4519264; doi:10.1371/journal.pone.0134408)

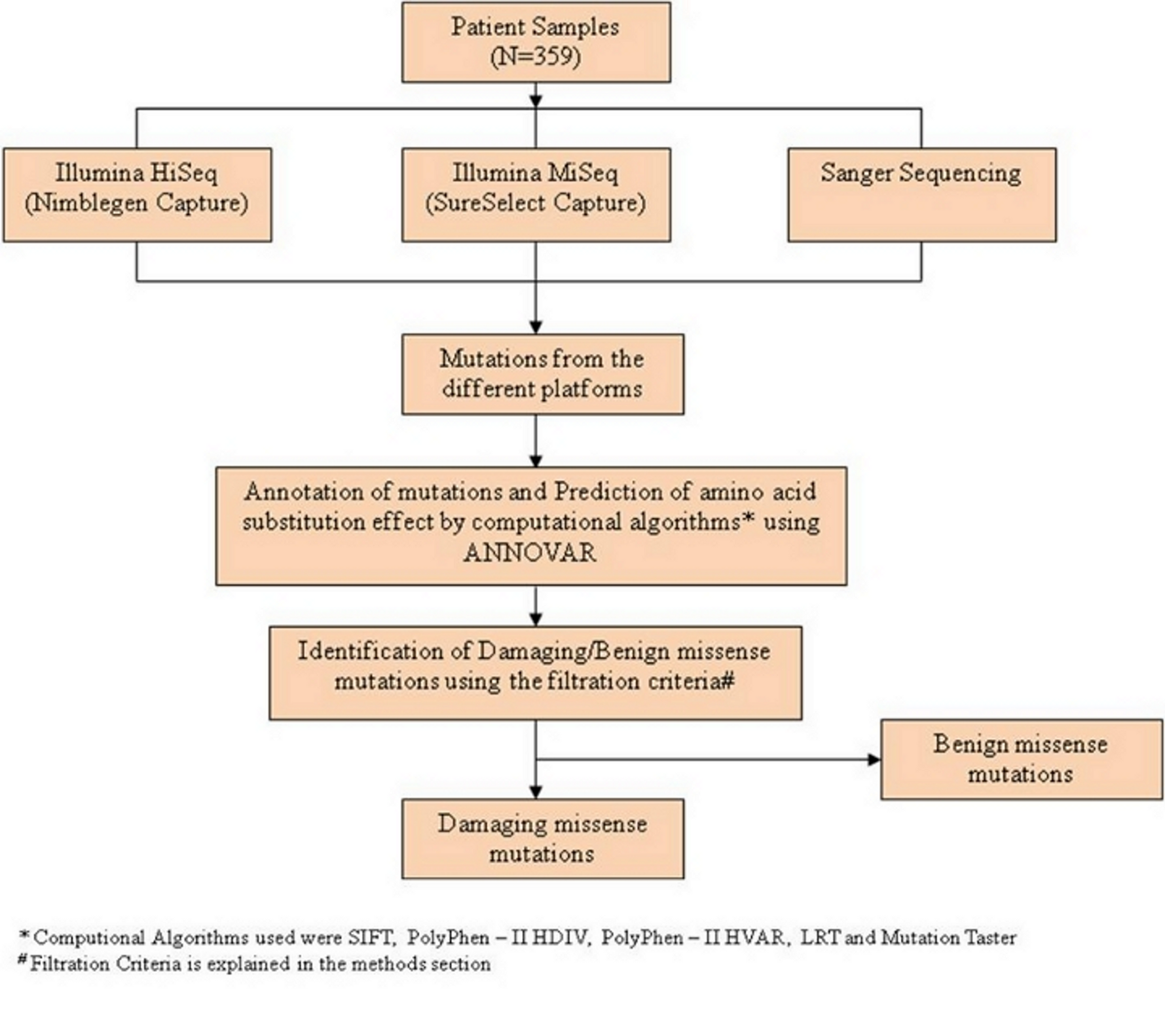

Supplement: S1 Fig — * Computational algorithms used were SIFT, Polyphen-II HDIV, Polyphen-II HVAR, LRT and Mutational Taster; # Filtration criteria is explained in the methods section. (TIF) [file pone.0134408.s001.tif]

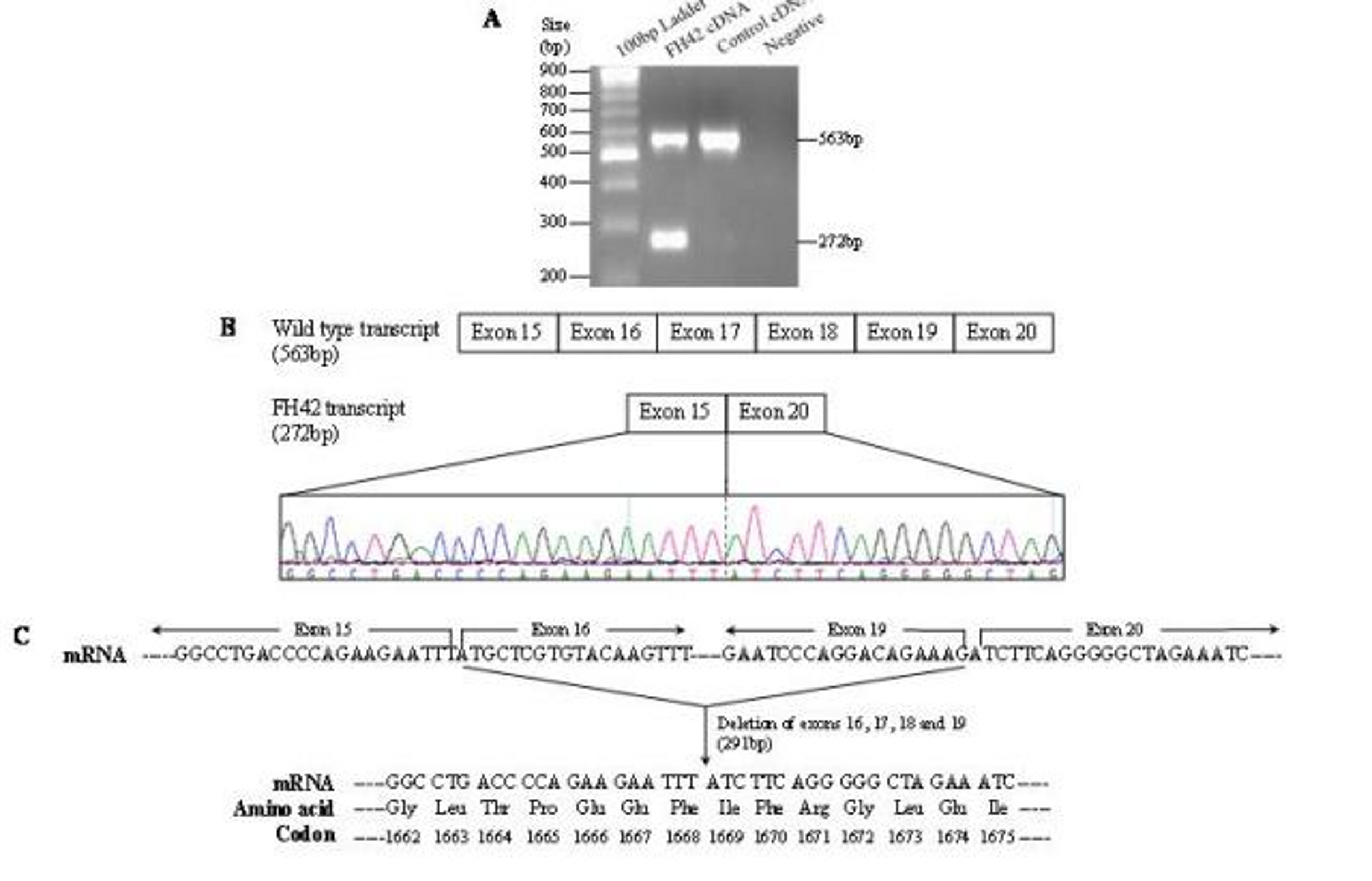

Supplement: S2 Fig — A) Gel photo of PCR products obtained from the amplification of a 563bp-target region from the sample FH42 and control cDNA template; and a sequencing chromatogram of the 272bp-band observed from FH42. (B and C) Changes in mRNA sequence brought about by the deletion of 291bp in FH42 and its corresponding amino acid sequence. (TIF) [file pone.0134408.s002.tif]
